# Preservation of essential oil quality in endangered *Ziziphora tenuior* L. under different storage conditions

**DOI:** 10.1038/s41598-025-24422-9

**Published:** 2025-11-18

**Authors:** Sharareh Najafian

**Affiliations:** Department of Natural Resources and Environment Engineering, School of Agriculture, Shiraz University, Shiraz, Iran

**Keywords:** Storage conditions, Labiatae, Pulegone, Industrial compounds, Biochemistry, Chemical ecology

## Abstract

*Ziziphora tenuior* L. is an endangered medicinal plant valued for its essential oil with strong antimicrobial and antioxidant properties. This study aimed to assess how storage temperature and duration influence the chemical composition of its essential oil. The oil was obtained from air-dried aerial parts via hydro distillation and stored for three months under three temperature conditions: room temperature (25 °C), refrigeration (4 °C), and freezing (-20 °C). Gas chromatography was used for compound identification, and the data were analyzed using Principal Component Analysis (PCA) and Selection Criteria (SC). Results showed significant degradation of low-boiling-point compounds at room temperature; notably, pulegone declined from 42.84% to 7.86%, and *cis*-carvone from 10.1% to 0.99%. In contrast, samples stored at freezing temperature retained a more stable chemical profile. These findings emphasize the importance of cold storage in preserving the chemical integrity and biological potential of essential oils, supporting their sustainable use in pharmaceutical, food, and health-related applications. To our knowledge, this is one of the first studies to comprehensively evaluate the time, temperature interaction on the stability of *Z. tenuior* essential oil. The outcomes can help establish standard postharvest practices for maintaining the quality of essential oils derived from endangered medicinal plants.

## Introduction

Medicinal plants are a rich source of bioactive secondary metabolites such as essential oils, phenolics, and flavonoids, widely recognized for their therapeutic, antimicrobial, and antioxidant properties^1,2^. These compounds, which serve as part of the plant’s defense mechanisms, also hold great economic and pharmaceutical value worldwide^3,4^. Among them, essential oils, volatile and aromatic in nature, are especially prone to degradation due to environmental and postharvest factors, which can significantly impact their chemical stability and bioactivity^5,6^. The biosynthesis and accumulation of these metabolites are influenced by environmental variables including temperature, soil conditions, and irrigation practices^7–9^. Postharvest handling, particularly storage temperature and duration, is therefore critical for preserving essential oil quality^5,10^. Inappropriate storage may lead to oxidation, volatilization, and breakdown of key constituents, diminishing their therapeutic efficacy and limiting their industrial applicability^11^.

**Fig. 1:**
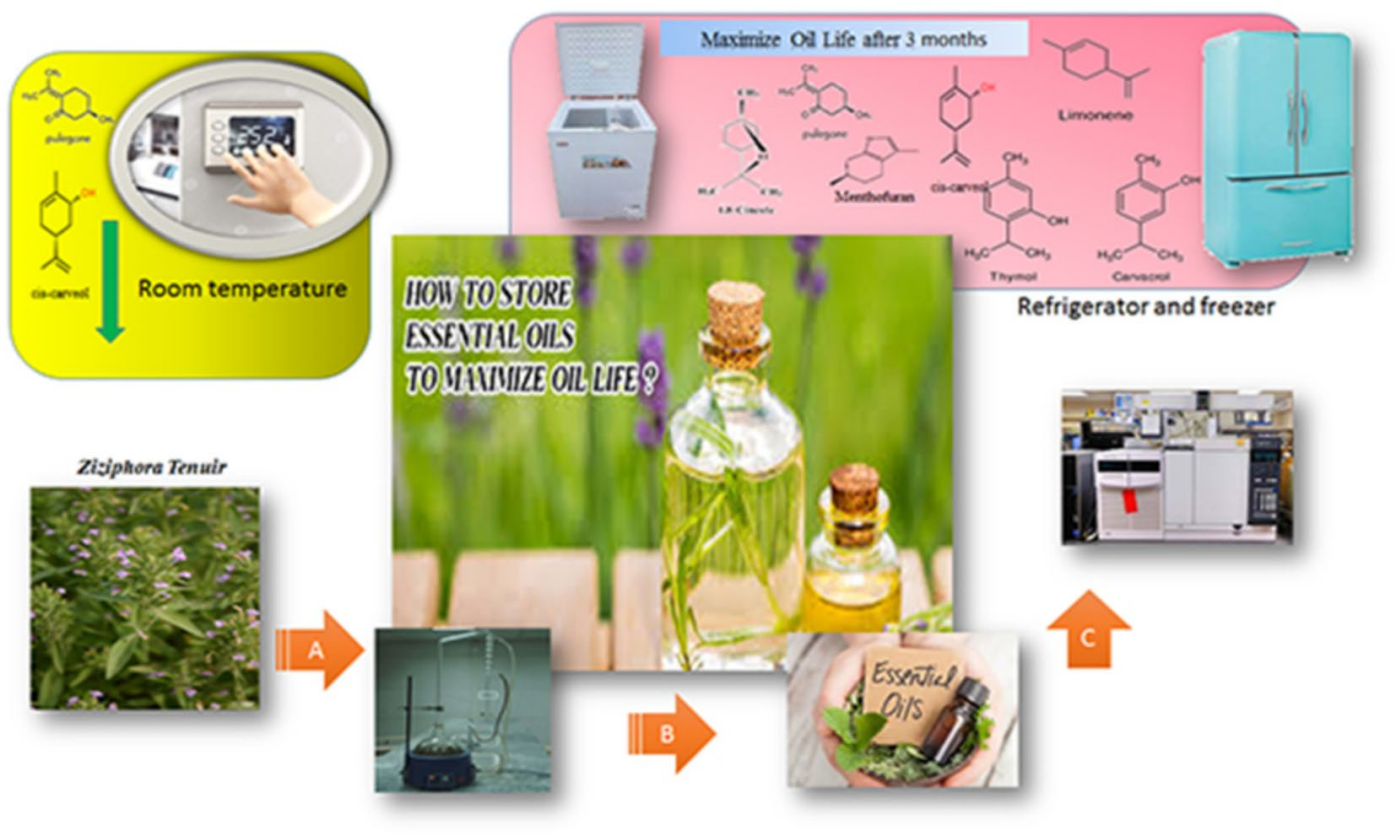
Investigating the storage life of *Z. tenuior* essential oil in different temperature conditions during three months of storage (This figure was originally created by the author using Adobe Photoshop).

*Ziziphora tenuior* L., a member of the Lamiaceae family, is an endangered medicinal plant native to Iran, known for its essential oil rich in compounds such as pulegone and cis-carvone with demonstrated antimicrobial and antioxidant activities^11,12^. Despite its ethnobotanical significance and wide potential for application in food, pharmaceutical, and cosmetic industries, limited research has addressed how storage conditions affect the chemical integrity of its essential oil, hindering the development of optimized postharvest handling protocols. Understanding the influence of storage temperature and duration is thus crucial for maintaining the functional and economic value of *Z. tenuior*. Recent studies on related medicinal plants, including *Salvia officinalis*, *Satureja hortensis*, and *Mentha* species, have emphasized the need for low-temperature storage to preserve essential oil composition and bioactivity over time^6,10^. Furthermore, the integration of advanced analytical techniques with multivariate statistical methods provides powerful tools to monitor chemical changes and develop effective preservation strategies^9,12^. This study aims to investigate the effects of different storage temperatures (room temperature, refrigeration, and freezing) and durations on the chemical composition of *Z. tenuior* essential oil. By monitoring compositional changes under various thermal conditions, the research seeks to propose effective, low cost, and sustainable strategies for preserving the bioactive stability and therapeutic efficacy of this endangered species. The findings are expected to inform the development of optimized postharvest practices that not only safeguard the medicinal value and enhance the industrial and economic potential of the plant but also contribute to the conservation and targeted utilization of underexplored yet valuable medicinal species such as *Z. tenuior* (Fig. 1). To the best of our knowledge, this is the first comprehensive study to evaluate the combined effects of storage temperature and duration on the essential oil profile of *Z. tenuior*, providing novel insights into maintaining its postharvest chemical integrity and biological functionality.

## Materials and methods

### Collecting plants and identifying

**Fig. 2:**
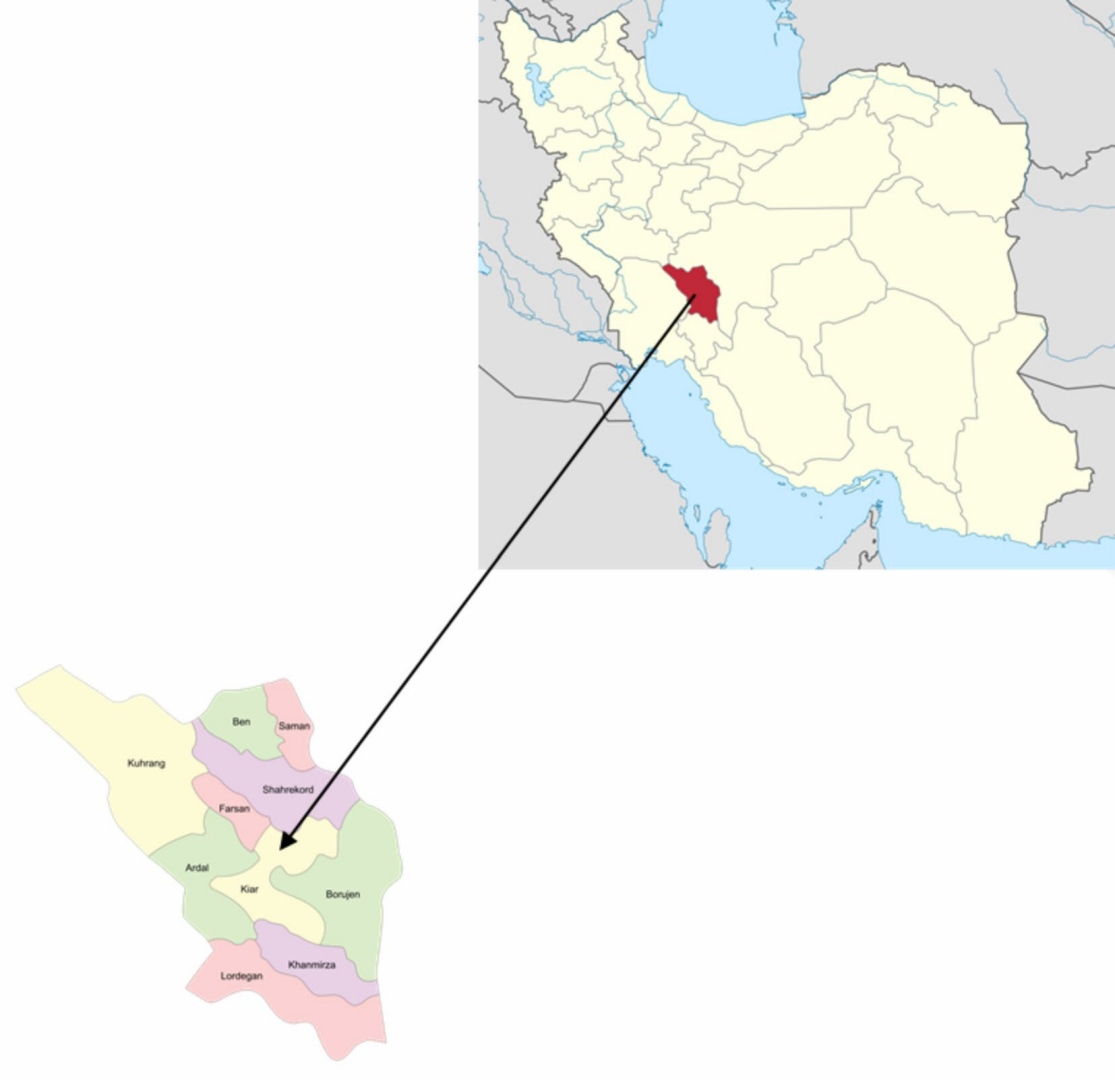
Sampling locations of wild populations of *Z. tenuior* in Chaharmahal and Bakhtiari province, Iran.

The city of Kiar in Chaharmahal and Bakhtiari Province, Iran (31°94’43” N, 50°72’3069” E) provided 1000 g samples of *Z. tenuior*. Ahmad Hatami (Faculty Member, Herbarium, Fars Research Center for Agriculture and Natural Resources, Shiraz, Iran) identified *Z. tenuior*. The herbarium now holds the voucher specimen (No. 15722). Local environmental laws and guidelines for the sustainable collection of medicinal herbs were followed during the collection. Non-destructive plant sample collection on public lands did not involve protected areas and no special permits were needed (Fig. 2). The samples were then recognized by Mr. Ahmad Hatami at the Fars Province Research Center (the voucher specimen: No. 16268). The aerial parts of the plants were collected in spring, on April 15, during the full flowering stage of the growing season, which is recognized as the optimal phase for essential oil accumulation. After harvest, the plant materials were carefully prepared for drying under suitable conditions. Pre-treatment included the removal of non-essential components and separation of primary parts to facilitate the drying process and enhance product quality. The samples were then shade-dried at room temperature (20–25 °C) for 14 days. Following the method suggested by the British Pharmacopoeia, the essential oil content of each dried specimen (100 g) was extracted by hydro-distillation at the boiling point of water (approximately 100 °C) for three hours using a Clevenger-type apparatus. The distilled essential oils were dried over anhydrous sodium sulfate and then stored in tightly sealed, dark vials to minimize exposure to oxygen and light. However, due to equipment limitations, anaerobic storage conditions were not employed^13^.

### The circumstances for storing volatile oils

In order to determine the precise effects of storage conditions on the compositions of the distilled essential oils, the prepared specimens were exposed to a range of storage temperatures for three consecutive months prior to examination: room temperature (25 °C), freezer (-20 °C), and refrigerator (4 °C). The essential oils were analyzed in each storage treatment on a monthly basis, and the fresh extracted essential oil was examined immediately after extraction to determine the precise effects of storage conditions on essential oil compositions throughout the test course. The extracted EOs were yellow and had a distinct and sharp smell^13^.

### GC and GC-MS analysis of essential oil

GC analysis was carried out utilizing an Agilent gas chromatograph series 7890-A with and FID (flame ionization detector). The detector’s temperatures were kept between 250 °C and 280 °C. As the carrier gas, nitrogen was used at a flow rate of 1 milliliter per minute. The oven was programmed to reach 240 °C at a rate of 20 °C per minute after first reaching 60–210 °C at a rate of 4 °C per minute. It was ultimately maintained at this temperature for 8.5 min. 1:50 was the split ratio. GC-MS and the analysis were carried out using an Agilent gas chromatograph equipped with a 5975-C mass spectrometer and a fused silica capillary HP-5MS column (0.25 mm × 30 mi.d., film thickness 0.25 m). The mass range was between 45 and 550 amu, the oven temperature program was similar to that stated for the GC, and helium was used as the carrier gas with an ionizing voltage of 70 eV. The interface and ion source temperatures were 280◦ C and 230◦ C, respectively^13^.

### Identifying the compounds

The components of the essential oils were identified by calculating their retention indices (RI) under temperature-programmed conditions using n-alkanes (C8–C25) on an HP-5 column under similar chromatographic conditions. The calculated RI values of the individual compounds were compared with those reported by Adams (2001) or with authentic reference standards. Additionally, the mass spectra of the compounds were evaluated using the internal spectral library or reference standards. The relative percentages of the components were determined using flame ionization detection (FID) without applying any correction factors^14^.

### Statistical design

Three replications and a factorial design based on a totally randomized design were used in this investigation. Three temperature levels (room temperature, refrigerator temperature (4 °C), and freezer temperature (-20 °C) made up Factor A, while storage times (one, two, and three months following essential oil extraction) made up Factor B.

### Statistical analysis

SAS 9.1.3 software was used for data analysis, Pearson correlation between the essential oil components of each plant was determined using SPSS 19 software, mean comparisons were performed using Duncan’s multiple range test at a 5% significance level, and graphs were created using Excel 2019 and GraphPad Prism 9.

## Results and discussion

### Chemical composition of essential oil of *Z. tenuior* at room temperature

Limited research has been conducted on the storage of plant secondary metabolites, particularly essential oils, which are volatile compounds that can undergo significant changes due to storage conditions. Researchers have proven that the time of harvest and storage conditions of essential oils have a significant impact on the quality and type of chemical composition of essential oils^15^. Researchers have demonstrated that high temperatures during the storage of medicinal plant essential oils cause evaporation and have a detrimental impact on their quality^16^. Because of the importance of the issue, as well as the storage and preservation of essential oils and their use in a variety of businesses, research on key medicinal plants has given promising results^17–20^.

**Fig. 3:**
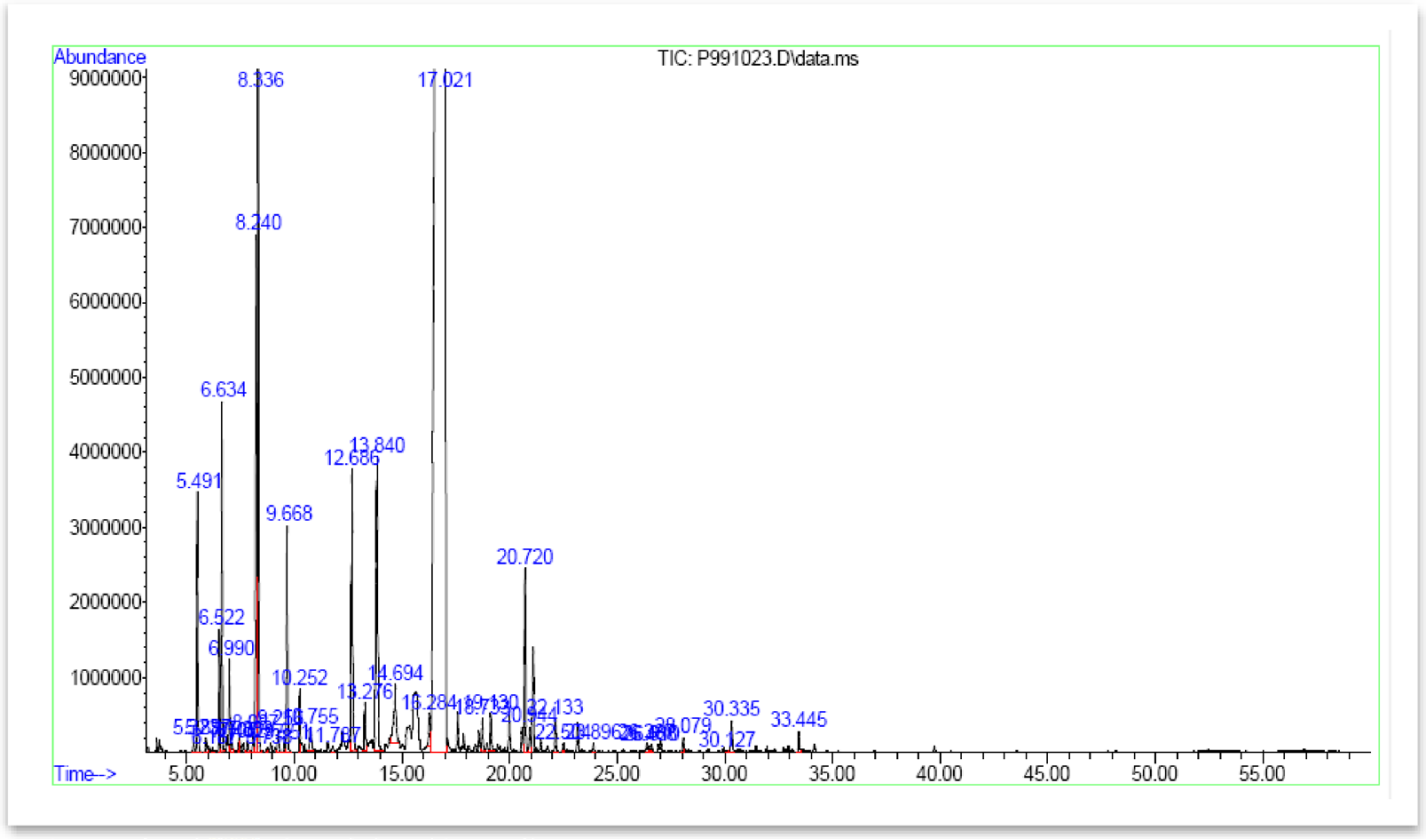
GC-MS chromatogram of the essential oil extracted from *Z. tenuior*, illustrating the relative abundance of volatile compounds plotted against their retention times. This profile allows precise identification and separation of the main constituents of the essential oil.

In the essential oil samples of *Z. tenuior*, 39 distinct components were identified and measured (Fig. 3). The monoterpenoid fraction accounted for 99.08% of the oil, with key constituents including β-Pinene, Limonene, 1,8-Cineole, *p*-Menth-3-en-8-ol, Neoiso-Isopulegol, Menthofuran, Pulegone, Thymol, and Carvacrol. The proportion of sesquiterpenes and sesquiterpenoid compounds was relatively minimal, at 0.27%. Monoterpenes are the predominant compounds in plant essential oils, and many of the therapeutic effects attributed to medicinal plants are linked to these compounds^21^.

A comparison of the essential oil composition under different storage temperatures and durations revealed significant alterations in the levels of the major components when stored at room temperature, in contrast to the corresponding storage conditions (Table 1).

**Table 1:** Composition of *Z. tenuior*’s hydro-distilled essential oils during 3 months storage at room temperature.

No	Compound%	RI_Exp_^*^	RI_Lit_*	RT***	After distillation (%)	After 1 month (%)	After 2 months (%)	After 3 months (%)
1	α-Thujene	926	926	5.32	0.01	0.02	0.0	0.0
2	α-Pinene	932	934	5.49	0.79	0.68	0.65	0.66
3	Camphene	949	948	5.92	0.04	0.10	0.21	-
4	Benzaldehyde	959	959	6.19	0.02	0.02	0.05	0.30
5	Sabinene	972	971	6.52	0.42	0.33	0.25	0.23
6	β-Pinene	976	976	6.63	1.29 ± 0.008a	1.14 ± 0.018a	1.08 ± 0.005a	1.08 ± 0.30a
7	Myrcene	990	990	6.99	0.41	0.20	0.15	-
8	3-Octanol	992	990	7.05	0.03	0.14	0.12	-
9	α-Phellandrene	1005	1005	7.43	0.04	0.04	0.05	-
10	α-Terpinene	1017	1015	7.83	0.01	0.02	0.03	-
11	*p*-Cymene	1024	1023	8.09	0.04	0.08	0.10	0.09
12	Limonene	1028	1027	8.24	2.36 ± 0.15a	2.09 ± 0.008a	2.06 ± 0.012a	1,97 ± 0.611a
13	1,8-Cineole	1031	1028	8.34	2.98 ± 0.005c	3.63 ± 0.020b	3.84 ± 0.034a	3.81 ± 0.003a
14	Benzene acetaldehyde	1042	1042	8.74	0.04	0.59	0.53	-
15	γ-Terpinene	1060	1056	9.36	0.10	0.01	0.01	0.36
16	*cis*-Sabinene hydrate	1070	1070	9.70	0.32	0.21	0.33	0.28
17	*p*-Mentha-3,8-diene	1072	1072	9.77	0.01	0.13	0.04	-
18	Terpinolene	1085	1088	10.25	0.07	0.03	0.06	-
19	Linalool	1098	1097	10.70	0.03	0.24	0.12	-
20	*α*-Campholenal	1125	1125	11.79	0.08	0.05	0.08	-
21	*p*-Menth-3-en-8-ol	1147	1147	12.69	1.96 ± 0.005b	1.98 ± 0.005b	2.19 ± 0.005a	0.0 ± 0.000c
22	Menthofuran	1162	1162	13.28	0.15 ± 0.01b	0.20 ± 0.005b	0.13 ± 0.017b	1.98 ± 0.34a
23	neoiso-Isopulegol	1175	1175	13.80	1.27	1.58	1.78	1.68
24	α-Terpineol	1190	1188	14.40	0.53	0.55	0.56	0.17
25	cis-Carveol	1230	1230	16.10	1.10 ± 0.031a	0.36 ± 0.018c	0.20 ± 0.012d	0.99 ± 0.005b
26	Pulegone	1237	1235	16.36	84.42 ± 0.18a	80.14 ± 0.059b	79.88 ± 0.33b	78.86 ± 0.40b
27	Thymol	1290	1291	18.60	0.15 ± 0.015b	0.62 ± 0.040b	2.78 ± 0.008a	0.54 ± 0.023b
28	Carvacrol	1299	1299	19.00	0.11 ± 0.0057d	2.43 ± 0.036b	0.44 ± 0.005c	2.95 ± 0.029a
29	Piperitenone	1340	1340	20.72	0.10	0.63	0.09	0.15
30	α-Copaene	1374	1376	22.13	0.09	0.10	0.10	0.63
31	β-Bourbonene	1383	1390	22.50	0.02	0.18	0.13	-
32	(E)-Caryophyllene	1417	1417	23.90	0.04	0.09	0.22	-
33	ar-Curcumene	1479	1479	26.40	0.05	0.14	0.05	-
34	Germacrene D	1481	1488	26.49	0.02	0.09	0.05	-
35	(E)-β-Ionone	1489	1491	27.24	0.01	0.07	0.15	-
36	δ-Cadinene	1519	1541	28.08	0.04	0.02	0.32	0.14
37	Spathulenol	1578	1580	30.13	0.01	0.01	-	-
38	Caryophyllene oxide	1583	1586	30.34	0.14	0.17	0.17	0.17
39	ar-Turmerone	1665	1665	33.45	0.08	0.08	-	-
	Total				99.35%	99.22%	98.97%	98.26%

Grouped components (%).

Monoterpene hydrocarbons (Sr. No. 1–3, 5–7,9–12, 15, 17, 18).

Oxygen-containing monoterpenes (Sr. No,4,8,13–14, 16, 19–29).

Sesquiterpene hydrocarbons (Sr. No. 30–35).

Oxygen-containing sesquiterpenes (Sr. No. 36–39).

Data are mean ± standard error of three replications. ND, not detected. RIExp*, retention indices. RI values were obtained from NIST database and Adams (2001); RILit**, Retention indices taken from literature (12, 37); RT***, Retention time; D, distillation (%); a, Means followed by the same letter within a row are not significantly different according to Duncan’s multiple range test at *P* < 0.05.

**Fig. 4:**
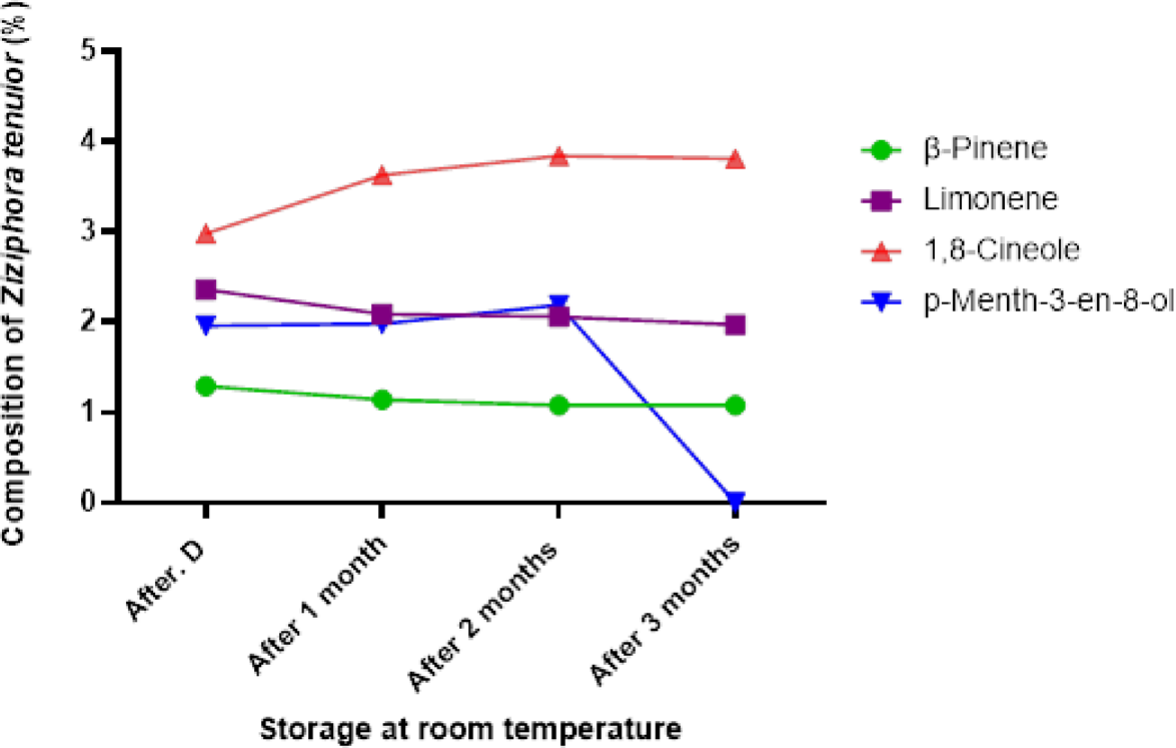
Investigating the storage life of *Z. tenuior* essential oil in room temperature conditions during three months of storage.

The results of our study show a consistent decline in the concentration of compounds with lower molecular weights during storage, particularly at room temperature (Table 1). This trend can be explained by their intrinsic physicochemical properties namely, their high vapor pressure, low boiling points, and high volatility. Such compounds, including pulegone and *cis*-carveol, are more prone to evaporation even at moderate temperatures. In addition, their chemical structures often contain reactive functional groups (e.g., double bonds or hydroxyl groups), making them more susceptible to oxidative degradation and polymerization when exposed to oxygen, heat, and light during storage. For instance, pulegone, a monoterpene ketone with a molecular weight of 152.23 g/mol, gradually decreased from 84.42% at extraction to 78.86% after 3 months. This loss is mainly due to evaporation and oxidative reactions forming degradation products such as menthofuran and isopulegone. Similarly, *cis*-carveol (MW: 152.23 g/mol), a monoterpenoid alcohol, exhibited a sharp initial decline due to its allylic hydroxyl group’s sensitivity to oxidation. These observations align with findings by Ganosi et al. (2023), who reported that even minor volatile constituents (< 1%) in essential oils like *Mentha spicata* and *Origanum vulgare* significantly decrease under ambient storage, while major components remain relatively stable^22^. Abdelmohsen and Elmaidomy (2025) further emphasize that environmental factors, particularly temperature, light, and oxygen, accelerate chemical changes such as oxidation, polymerization, and hydrolysis, compromising the stability of volatile monoterpenes and other low-molecular-weight compounds. Therefore, the decline in these compounds is a direct consequence of their physicochemical instability and exposure to suboptimal storage conditions, rather than mere assumptions. These chemical transformations are well documented in the literature and are accelerated by elevated temperature and light exposure, disrupting the stability of these volatile compounds^23^. The quality and biological activities of essential oils are known to be influenced by various factors, including extraction methods, environmental conditions, and storage parameters. Our findings align with several recent studies that emphasize the multifaceted nature of these influences. Karimnejad and Ghavam (2024)^5^ demonstrated that the extraction technique plays a pivotal role in determining the yield, chemical composition, and antimicrobial efficacy of essential oils. Their comprehensive comparison of traditional and modern extraction methods on *Mentha longifolia* showed that hydro distillation (HDC) provided the highest oil yield, while steam distillation (SDK) yielded the most diverse chemical profile. Furthermore, methods such as hydrodistillation with microwave (HDM) and ultrasonic pretreatment combined with hydrodistillation (U + HDC) exhibited enhanced antimicrobial activities. These findings suggest that selecting an appropriate extraction method is critical not only for maximizing oil yield but also for optimizing the spectrum of bioactive compounds, which in turn can affect the oil’s stability and therapeutic potential during storage. Complementing this perspective, Hosnaroodi and Ghavam (2025)^6^ highlighted the significant impact of environmental factors, specifically soil and irrigation water characteristics, on the composition, yield, and antimicrobial activity of *Mentha spicata* essential oil collected from different regions. Their study found substantial variation in essential oil profiles and bioactivities attributable to geographic and edaphic differences. This underscores the importance of considering pre-harvest environmental conditions in evaluating essential oil quality, which may also influence the oils’ susceptibility to chemical changes during storage. Moreover, Ghavam (2022)^2^ reviewed the influence of storage conditions on essential oils, emphasizing that factors such as temperature, light exposure, and oxygen availability critically determine the chemical stability and biological efficacy of essential oils over time. Specifically, volatile compounds with lower molecular weights tend to diminish due to evaporation and oxidation, particularly when stored at room temperature. In contrast, certain more stable or transformation-prone compounds may increase in relative concentration during storage through processes like isomerization or cyclization. Our study on *Z. tenuior* corroborates these findings by demonstrating significant compositional shifts dependent on storage temperature and duration. For instance, compounds like pulegone decreased substantially over time at room temperature, whereas compounds such as 1,8-cineole and carvacrol exhibited concentration increases, potentially due to chemical transformations occurring during storage. Collectively, these studies illustrate that the bioactivity and chemical integrity of essential oils are governed by a complex interplay of factors spanning from plant growth environment and harvesting, through extraction methodology, to storage conditions. Our results highlight the necessity of integrating these considerations to ensure the preservation of essential oil quality and efficacy. Such an integrative approach is crucial for advancing the development of reliable herbal medicines and natural antimicrobial agents derived from Lamiaceae family plants. In this study, the storage temperature had a significant impact on the primary components of *Z. tenuior* essential oil. A previous investigation on *Melissa officinalis* L. revealed that key constituents such as citronellal, neral, and geranial exhibited a decrease under storage conditions, with the greatest reduction observed at room temperature and the least at freezer temperature^17^. It has been suggested in earlier studies that the changes in essential oil components during storage, such as evaporation and oxidation, could be linked to the molecular weight of the compounds^13^. Conversely, in this study, certain components, including menthofuran, 1,8-cineole, thymol, and carvacrol, showed an increase in concentration over time, particularly when stored at room temperature. At the time of distillation, 1,8-cineole was present at 2.98%, and after three months of storage, its concentration increased to 3.81%. The percentage of 1,8-cineole followed an ascending pattern: 2.98%, 3.63%, 3.84%, and 3.81% at distillation, and after 1, 2, and 3 months of storage at room temperature, respectively (Table 1; Fig. 4). A similar pattern was observed for carvacrol. Initially, carvacrol was 0.11% during oil extraction, and after 1, 2, and 3 months of storage, its content reached 2.43%, 0.44%, and 2.95%, respectively. These fluctuations in the composition and concentration of essential oil components can be attributed to various factors, such as evaporation and oxidation during storage^17^. Additionally, essential oils may undergo transformations into other phytochemicals through chemical reactions like isomerization, dehydrogenation, and cyclization^13,17^. Consequently, less stable compounds tend to degrade more rapidly due to their chemical interactions with other constituents^14^, which occur over time and are influenced by storage conditions.

### The main medicinal compounds of *Z. tenuior* under the influence of storage time and room temperature based on PCA

Principal Component Analysis (PCA) is an eigenvector-based technique primarily used to minimize data complexity by reducing the number of variables. This method converts the original variables into a smaller set of principal components that account for most of the data’s variance. PCA aims to resolve data conflicts by organizing multiple variables into distinct components, with the first component capturing the highest possible variance, followed by additional components that represent decreasing levels of variance. In this study, component rotation was performed using Prism 9 software. The key compounds in each principal component were determined based on the selection criterion (SC), calculated using a specific formula^24^.

$${\text{SC}} = \frac{{0.5}}{{\left( {{\text{PC}}_{{{\text{Eigenvalue}}}} } \right)^{{0.5}} }}$$

**Fig. 5:**
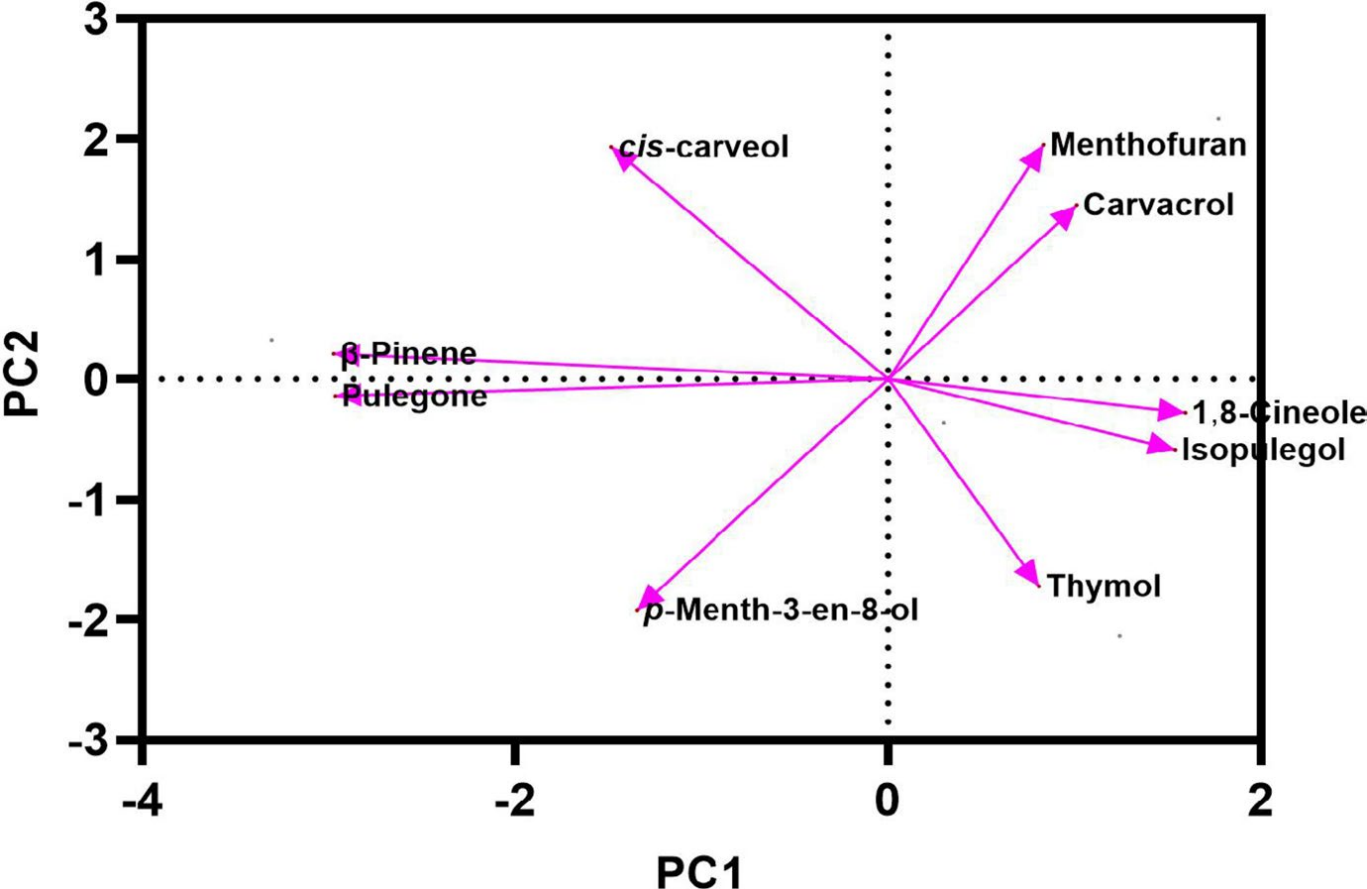
Principal component analysis (PCA) based on the main essential oil constituents of *Z. tenuior* stored at room temperature for three months. To enhance readability, only compounds with high relative percentages were included.

**Fig. 6:**
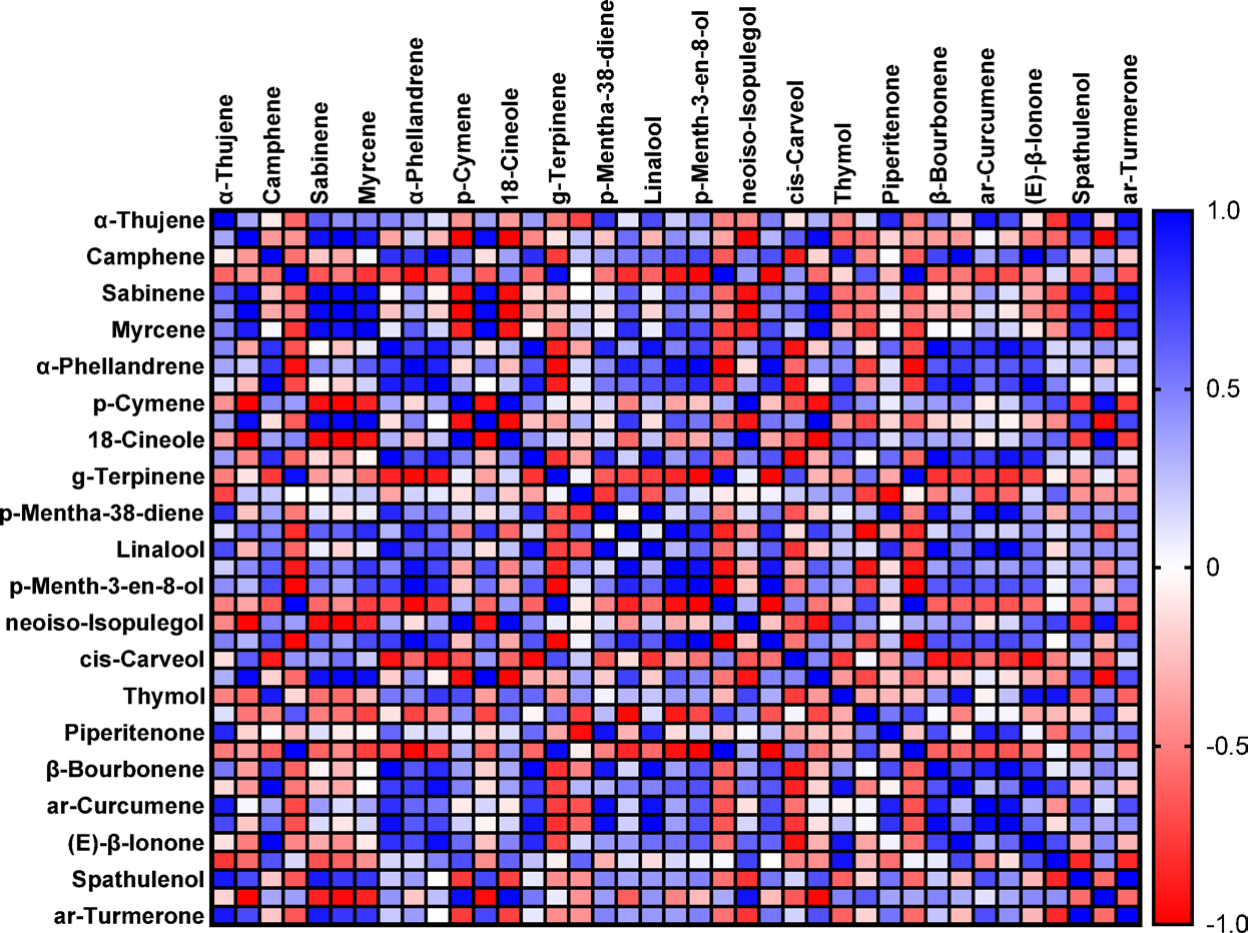
The correlation heat map of major compounds of essential oil (EO) of *Z. tenuior at* room temperature for three months.

Table 2 Presents the principal component analysis (PCA) outcomes, highlighting three primary factors with eigenvalues above 1 (Eigenvalue ≥ 1). Each variable within these main components received a weight based on its contribution to essential oil compounds, the higher the contribution, the greater the assigned weight. The significance of the compound’s role increases proportionally with the weight assigned. Following the preliminary screening of indicators, three key components demonstrated eigenvalues ≥ 1 and accounted for a cumulative variance of 100% under room temperature conditions over a three-month storage period (Table 2). The relative variance of each factor indicates its significance within the total variance of the assessed traits and is presented as a percentage. As shown in Table 2, three main and distinct components with eigenvalues exceeding one accounted for 100% of the total variance. In the first component (PC1), *p*-Menth-3-en-8-ol (0.235) with a positive coefficient and menthofuran (-0.237) with a negative coefficient stood out, collectively representing 44.31% of the total variance. For the second component (PC2), compounds such as β-Pinene (0.236), limonene (0.210), 1,8-Cineole (0.210), *cis*-Carveol (0.229), and pulegone (0.220) with positive coefficients explained up to 81.14% of the total variance. In the third component (PC3), thymol (-0.222) with a negative coefficient and carvacrol (0.275) with a positive coefficient accounted for 100% of the variance. This PCA analysis explored the association between the medicinal compounds in the essential oil and storage at room temperature. As depicted in Fig. 5, the proportion of therapeutic compounds was 44.31% for PC1 and 36.83% for PC2 (Fig. 5). Additionally, within each principal component (PC), a small angle and proximity between two compounds indicate a positive correlation between them. In addition to the results presented, Fig. 6 displays a pearson correlation heat map illustrating the relationship between the contents and identified compounds of *Z. tenuior* essential oil stored at room temperature.

**Table 2:** Principle component analysis (PCA) of essential oil components of *Z. tenuior* at room temperature for three months.

Principle components	PC1	PC2	PC3
Eigenvalue	17.28	14.36	7.35
Variability %	44.31	36.83	18.86
Cumulative	44.31	81.14	100.00
Selection criterion (SC)	0.120	0.131	0.184
	Eigen vectors		
Pinene	0.108	0.236	-0.009
Limonene	0.137	0.210	-0.073
1,8- Cineole	0.137	0.210	-0.073
*p*-Menth-3-en-8-ol	0.235	-0.018	-0.072
Menthofuran	-0.237	-0.002	0.059
*cis*-Carveol	-0.119	0.229	-0.001
Pulegone	0.121	0.220	-0.084
Thymol	0.048	-0.204	-0.222
Carvacrol	-0.1444	-0.078	0.275

^a^Boldface factor loading is considered highly weighted.

### Chemical composition of essential oil of *Z. tenuior* at refrigerator and freezer

**Fig. 7:**
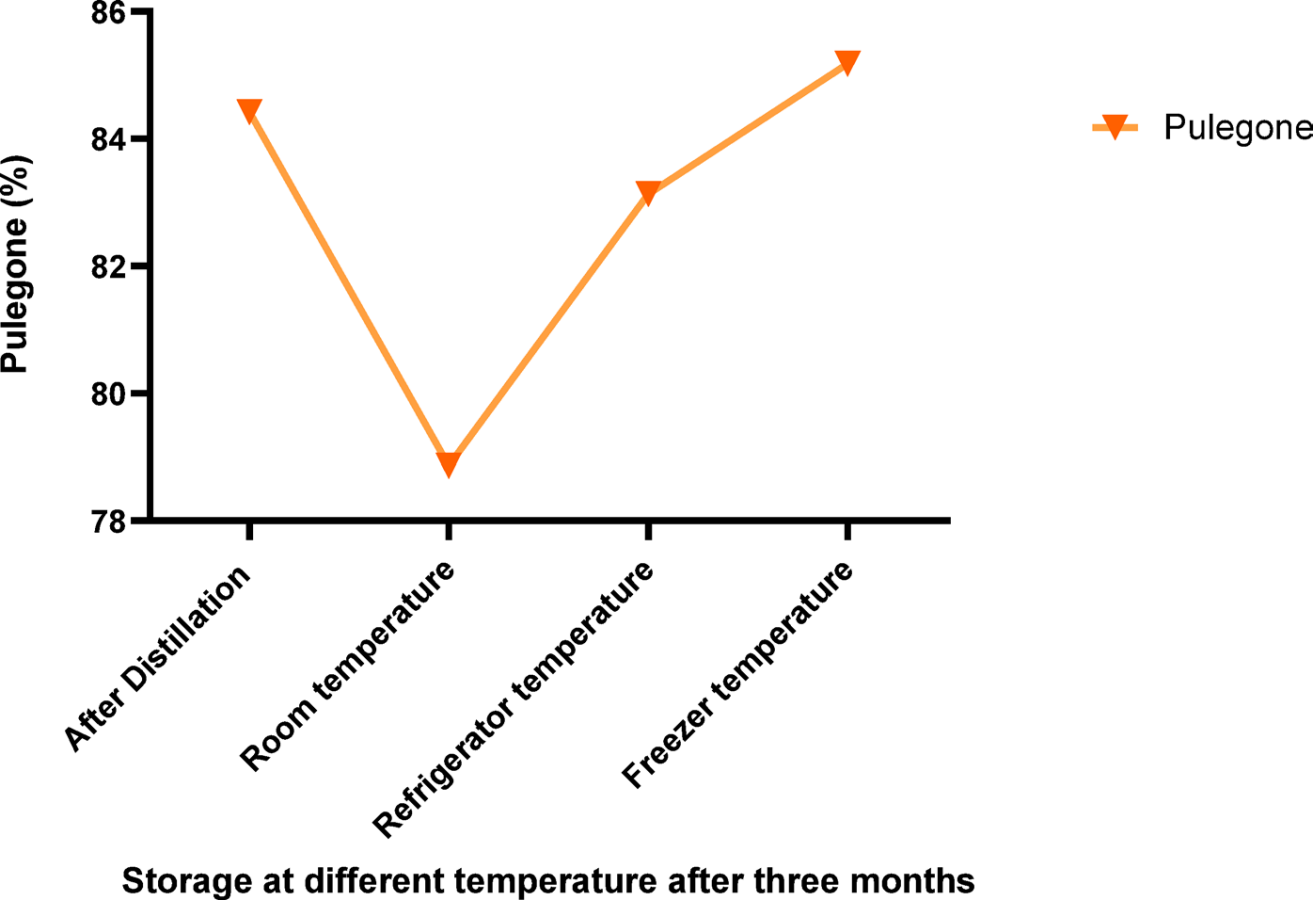
Percentage of pulegone of *Z. tenuior* essential oils after three months of storage at room temperature (25 °C), refrigerator (4 °C), and freezer (− 20 °C) conditions.

**Fig. 8:**
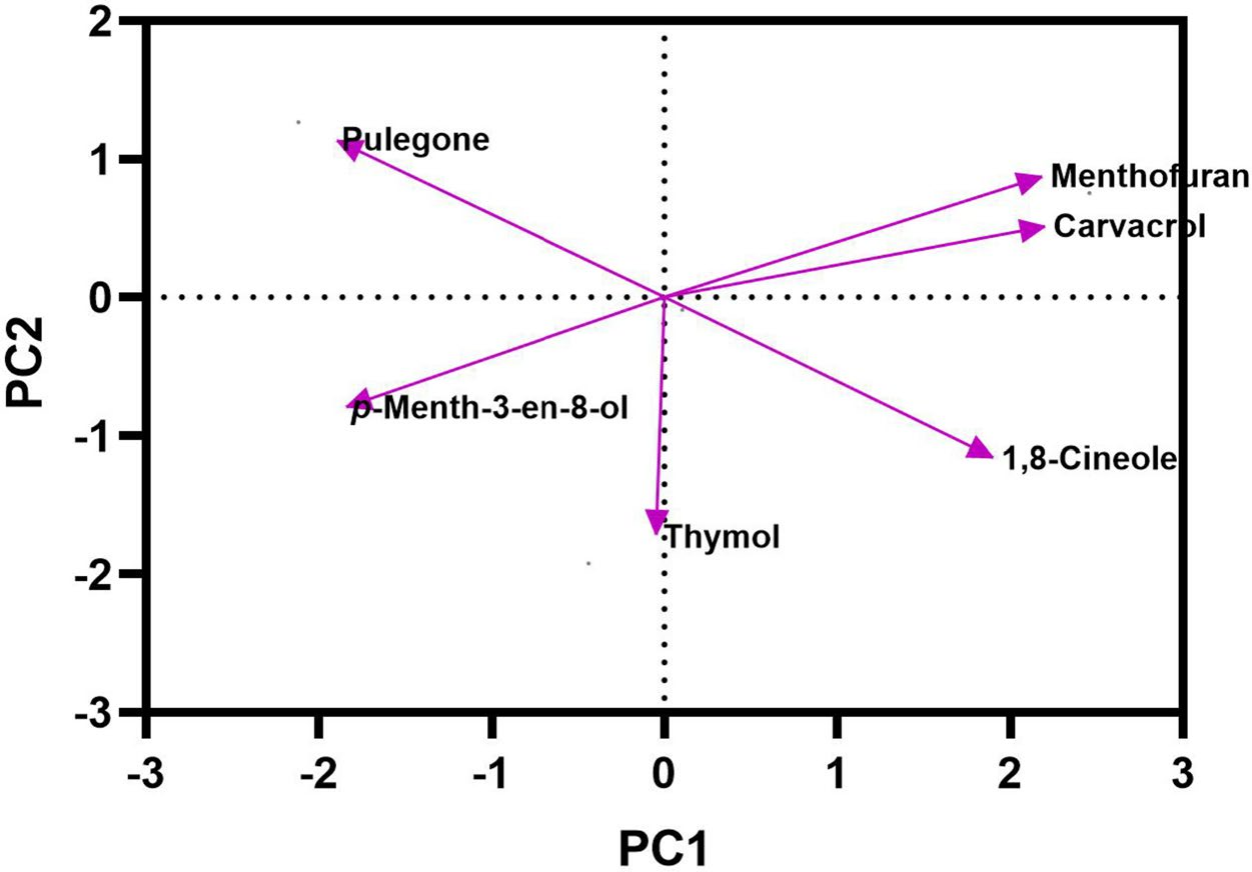
Principal component analysis (PCA) based on the main essential oil constituents of *Z. tenuior* stored at refrigerator (4℃) temperature for three months. To enhance readability, only compounds with high relative percentages were included.

**Fig. 9:**
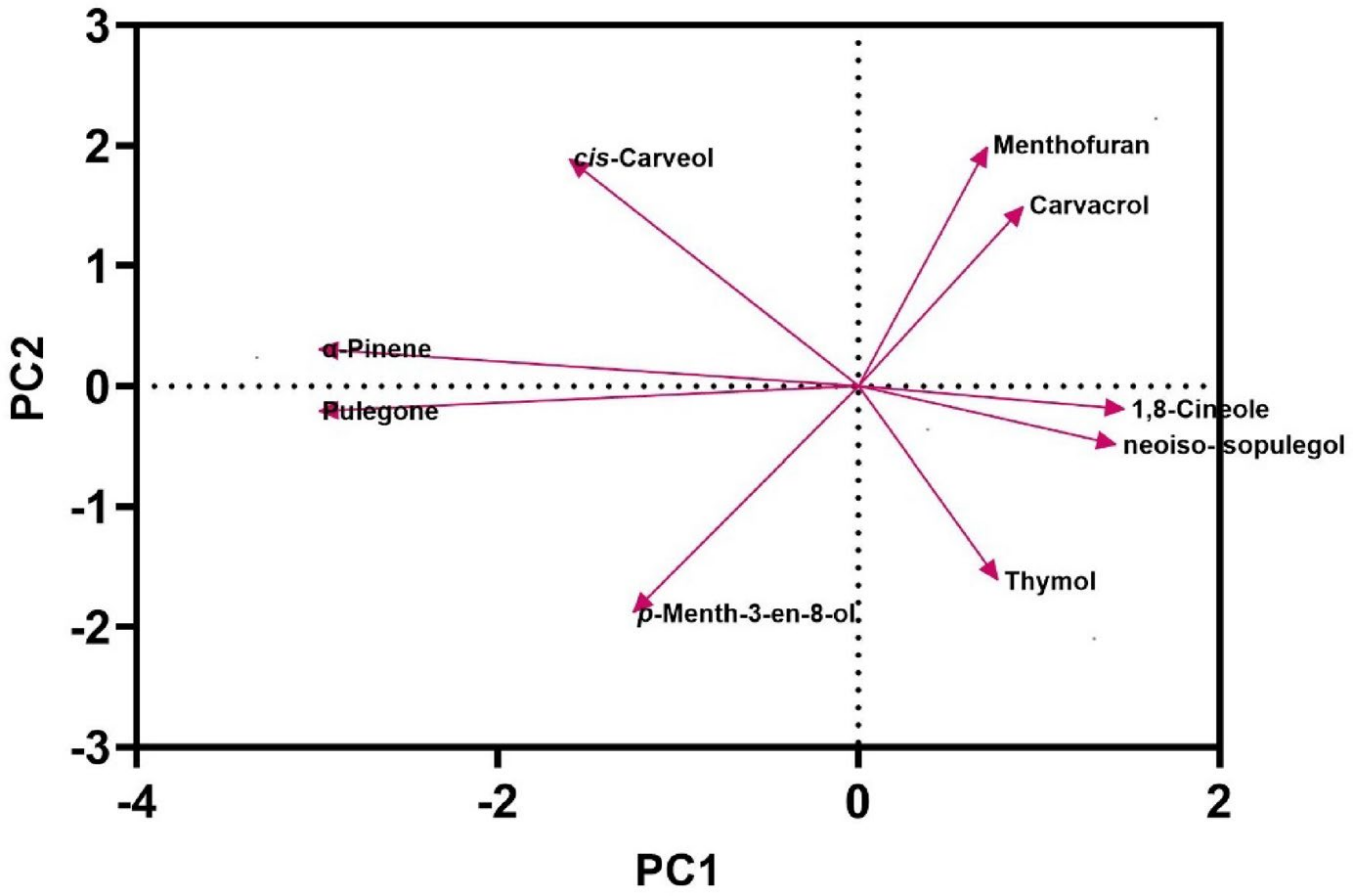
Principal component analysis (PCA) based on the main essential oil constituents of *Z. tenuior* stored at freezer temperature for three months. To enhance readability, only compounds with high relative percentages were included.

**Fig. 10:**
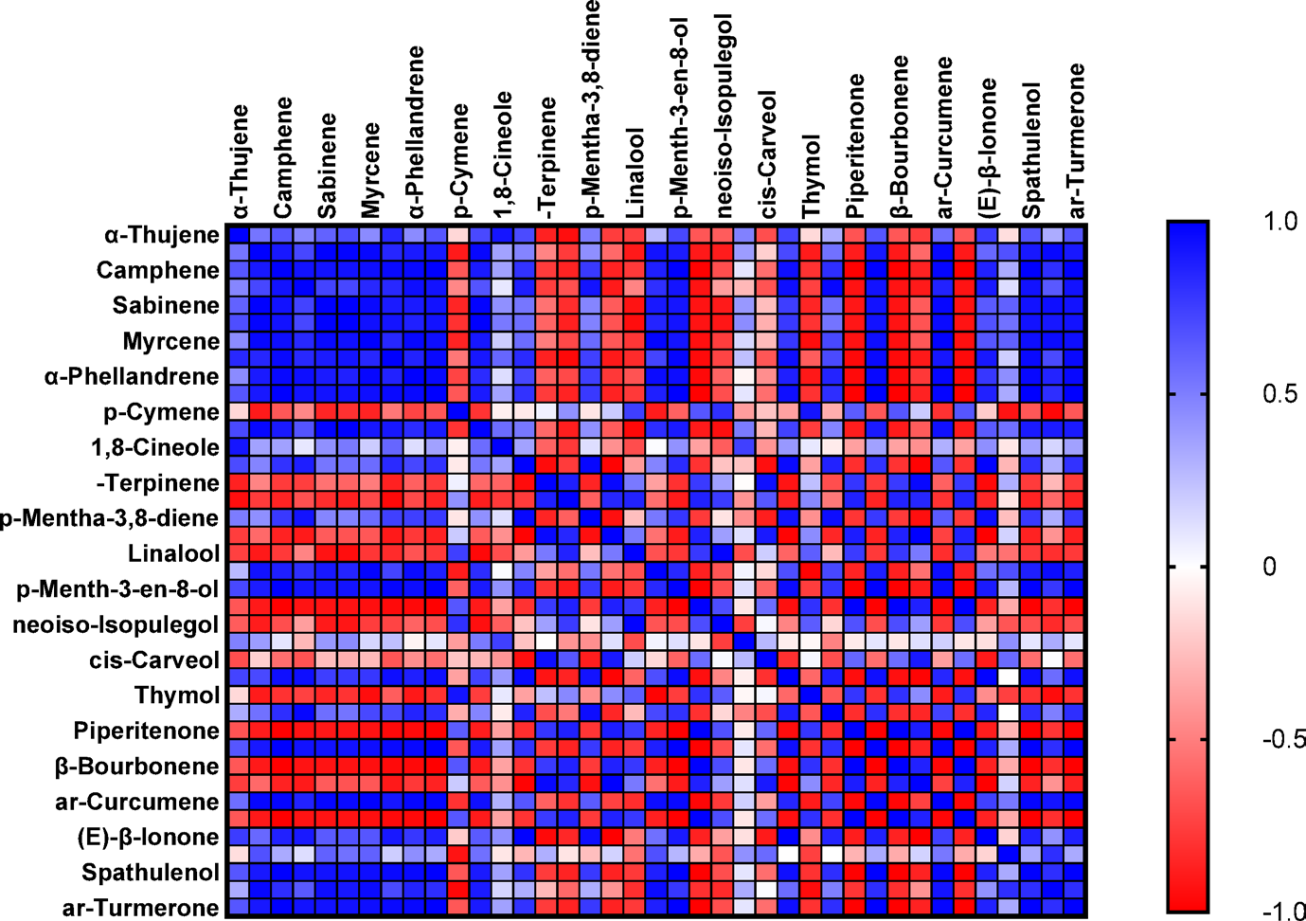
The correlation heat map of major compounds of essential oil (EO) of *Z. tenuior* at refrigerator (4℃) temperature for three months.

**Fig. 11:**
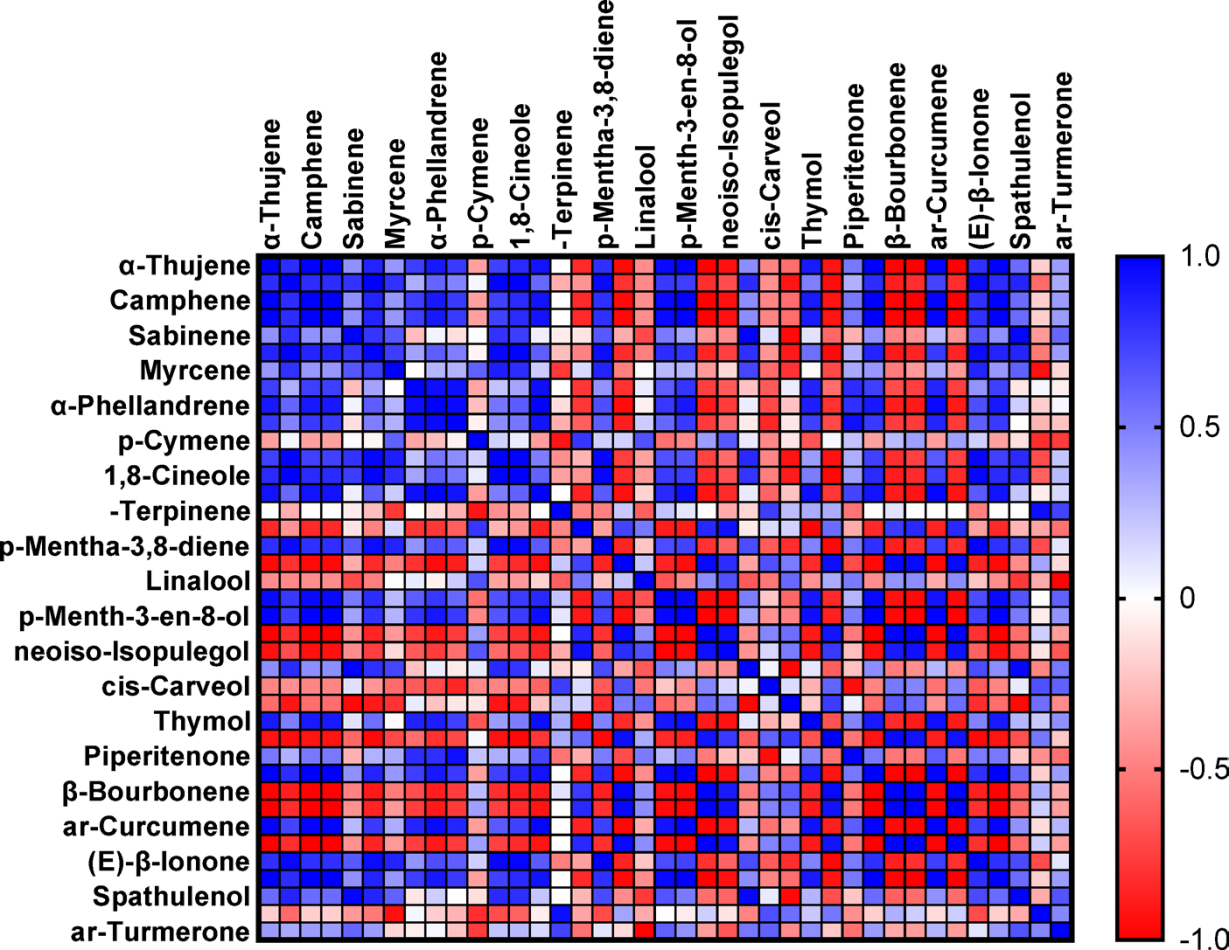
The correlation heat map of major compounds of essential oil (EO) of *Z. tenuior* at freezer temperature for three months.

The results indicated that essential oils stored at refrigeration (4 °C) and freezing (− 20 °C) temperatures exhibited the least degradation of their primary components, particularly pulegone, when compared to storage at room temperature. After three months of storage, these two temperature conditions (4 °C and − 20 °C) retained the highest concentration of pulegone (Fig. 7 Tables 3 and 4). In addition to pulegone, other key compounds such as β-Pinene, limonene, 1,8-cineole, thymol, and carvacrol also maintained their stability under refrigeration and freezing conditions (Tables 3 and 4). Figures 8 and 9 illustrate the distribution of therapeutic compounds in the plants based on the first and second principal components (PC1 and PC2) under room temperature, refrigeration, and freezing conditions. When the two pathways of a given compound are closely aligned and form a small angle, a positive correlation between the compounds is suggested. Furthermore, the Pearson correlation heatmap for the essential oil composition and the identified compounds stored at freezing and refrigeration temperatures for *Z. tenuior* essential oil is presented in Figs. 10 and 11.

**Table 3:** Composition of *Z. tenuior*’s hydro-distilled essential oils during 3 months storage at refrigerator temperature.

No	Compound%	RI_Exp_*	RI_Lit_**	RT***	After distillation (%)	After 1 month (%)	After 2 months (%)	After 3 months (%)
1	α-Thujene	926	926	5.32	0.01	0.01	0.03	
2	α-Pinene	932	934	5.49	0.79	0.76	0.77	0.72
3	Camphene	949	948	5.92	0.04	0.04	0.04	
4	Benzaldehyde	959	959	6.19	0.02	0.03	0.02	
5	Sabinene	972	971	6.52	0.42	0.40	0.41	0.37
6	β-Pinene	976	976	6.63	1.29 ± 0.008a	1.25 ± 0.005a	1.28 ± 0.005a	1.19 ± 0.34a
7	Myrcene	990	990	6.99	0.41	0.38	0.37	0.29
8	3-Octanol	992	990	7.05	0.03	0.03	0.04	
9	α-Phellandrene	1005	1005	7.43	0.04	0.04	0.03	
10	α-Terpinene	1017	1015	7.83	0.01	0.01	0.01	
11	p-Cymene	1024	1023	8.09	0.04	0.06	0.06	0.07
12	Limonene	1028	1027	8.24	2.36 ± 0.012a	2.30 ± 0.012a	2.35 ± 0.014a	2.23 ± 0.35a
13	1,8-Cineole	1031	1028	8.34	2.98 ± 0.005a	2.95 ± 0.008a	3.06 ± 0.008a	2.96 ± 0.003a
14	Benzene acetaldehyde	1042	1042	8.74	0.04	0.11	0.10	
15	γ-Terpinene	1060	1056	9.36	0.10	0.04	0.01	0.14
16	cis-Sabinene hydrate	1070	1070	9.70	0.32	0.34	0.20	0.50
17	p-Mentha-3,8-diene	1072	1072	9.77	0.01	0.03	0.02	
18	Terpinolene	1085	1088	10.25	0.07	0.03	0.03	0.11
19	Linalool	1098	1097	10.70	0.03	0.17	0.03	0.24
20	α-Campholenal	1125	1125	11.79	0.08	0.06	0.04	
21	p-Menth-3-en-8-ol	1147	1147	12.69	1.96 ± 0.006a	2.01 ± 0.005a	2.14 ± 0.005a	0.0 ± 0.008b
22	Menthofuran	1162	1162	13.28	0.15	0.15	0.14	1.78
23	neoiso-Isopulegol	1175	1175	13.80	1.27	1.48	1.31	1.53
24	α-Terpineol	1190	1188	14.40	0.53	0.33	0.55	0.45
25	cis-Carveol	1230	1230	16.10	1.10	0.28	0.26	1.09
26	Pulegone	1237	1235	16.36	84.42 ± 0.008a	85.11 ± 0.008a	85.05 ± 0.005a	83.13 ± 0.008a
27	Thymol	1290	1291	18.60	0.15 ± 0.005a	0.28 ± 0.005a	0.41 ± 0.003a	0.57 ± 0.19a
28	Carvacrol	1299	1299	19.00	0.11 ± 0.005a	0.12 ± 0.003a	0.11 ± 0.003a	0.10 ± 0.951a
29	Piperitenone	1340	1340	20.72	0.10	0.08	0.08	0.62
30	α-Copaene	1374	1376	22.13	0.09	0.09	0.09	
31	β-Bourbonene	1383	1390	22.50	0.02	0.02	0.02	0.08
32	(E)-Caryophyllene	1417	1417	23.90	0.04	0.03	0.02	0.05
33	ar-Curcumene	1479	1479	26.40	0.05	0.04	0.04	-
34	Germacrene D	1481	1488	26.49	0.02	0.02	0.02	0.04
35	(E)-β-Ionone	1489	1491	27.24	0.01	0.02	0.02	-
36	δ-Cadinene	1519	1541	28.08	0.04	0.03	0.04	0.03
37	Spathulenol	1578	1580	30.13	0.01	0.01	-	-
38	Caryophyllene oxide	1583	1586	30.34	0.14	0.13	0.15	0.12
39	ar-Turmerone	1665	1665	33.45	0.08	0.08	-	-
	Total				99.35%	99.36%	99.35%	99.12%

Grouped components (%).

Monoterpene hydrocarbons (Sr. No. 1–3, 5–7,9–12, 15, 17, 18).

Oxygen-containing monoterpenes (Sr. No,4,8,13–14, 16, 19–29).

Sesquiterpene hydrocarbons (Sr. No. 30–35).

Oxygen-containing sesquiterpenes (Sr. No. 36–39).

Data are mean ± standard error of three replications. ND, not detected. RIExp*, retention indices. RI values were obtained from NIST database and Adams (2001); RILit**, Retention indices taken from literature (12, 37); RT***, Retention time; D, distillation (%); a, Means followed by the same letter within a row are not significantly different according to Duncan’s multiple range test at *P* < 0.05.

**Table 4:** Composition of *Z. tenuior*’s hydro-distilled essential oils during 3 months storage at freezer temperature.

No	Compound%	RI_Exp_*	RI_Lit_**	RT***	After distillation (%)	After 1 month (%)	After 2 months (%)	After 3 months (%)
1	α-Thujene	926	926	5.32	0.01	0.01	0.01	
2	α-Pinene	932	934	5.49	0.79	0.83	0.76	0.71
3	Camphene	949	948	5.92	0.04	0.04	0.04	
4	Benzaldehyde	959	959	6.19	0.02	0.02	0.02	
5	Sabinene	972	971	6.52	0.42	0.43	0.33	0.35
6	β-Pinene	976	976	6.63	1.29 ± 0.015 a	1.33 ± 0.005a	1.25 ± 0.031a	1.18 ± 0.005a
7	Myrcene	990	990	6.99	0.41	0.92	0.40	0.36
8	3-Octanol	992	990	7.05	0.03	0.03	0.07	
9	α-Phellandrene	1005	1005	7.43	0.04	0.05	0.07	
10	α-Terpinene	1017	1015	7.83	0.01	0.02	0.03	
11	p-Cymene	1024	1023	8.09	0.04	0.09	0.06	0.08
12	Limonene	1028	1027	8.24	2.36 ± 0.005a	2.47 ± 0.005a	2.31 ± 0.031a	2.23 ± 0.017a
13	1,8-Cineole	1031	1028	8.34	2.98 ± 0.030a	3.09 ± 0.028a	2.93 ± 0.012a	2.80 ± 0.017a
14	Benzene acetaldehyde	1042	1042	8.74	0.04	0.04	0.06	
15	γ-Terpinene	1060	1056	9.36	0.10	0.02	0.06	0.06
16	cis-Sabinene hydrate	1070	1070	9.70	0.32	0.59	0.34	0.80
17	p-Mentha-3,8-diene	1072	1072	9.77	0.01	0.02	0.01	
18	Terpinolene	1085	1088	10.25	0.07	0.03	0.03	0.19
19	Linalool	1098	1097	10.70	0.03	0.11	0.14	0.14
20	α-Campholenal	1125	1125	11.79	0.08	0.06	0.06	
21	p-Menth-3-en-8-ol	1147	1147	12.69	1.96 ± 0.029a	1.74 ± 0.017b	1.98 ± 0.030a	0.08 ± 0.015c
22	Menthofuran	1162	1162	13.28	0.15 ± 0.005b	0.16 ± 0.005b	0.15 ± 0.015b	1.49 ± 0. 255a
23	neoiso-Isopulegol	1175	1175	13.80	1.27	1.44	1.39	1.75
24	α-Terpineol	1190	1188	14.40	0.53	0.60	0.25	0.31
25	cis-Carveol	1230	1230	16.10	1.10	0.16	0.16	0.96
26	Pulegone	1237	1235	16.36	84.42 ± 0.46a	84.07 ± 0.025a	85.15 ± 0.311a	85.18 ± 0.020a
27	Thymol	1290	1291	18.60	0.15 ± 0.005a	0.11 ± 0.005a	0.16 ± 0.005a	0.05 ± 0.76a
28	Carvacrol	1299	1299	19.00	0.11 ± 0.005a	0.08 ± 0.005a	0.11 ± 0.008a	0.17 ± 0.048a
29	Piperitenone	1340	1340	20.72	0.10	0.44	0.62	0.12
30	α-Copaene	1374	1376	22.13	0.09	0.09	0.09	0.04
31	β-Bourbonene	1383	1390	22.50	0.02	0.01	0.02	0.09
32	(E)-Caryophyllene	1417	1417	23.90	0.04	0.04	0.04	0.09
33	ar-Curcumene	1479	1479	26.40	0.05	0.05	0.06	-
34	Germacrene D	1481	1488	26.49	0.02	0.02	0.02	0.04
35	(E)-β-Ionone	1489	1491	27.24	0.01	0.02	0.01	-
36	δ-Cadinene	1519	1541	28.08	0.04	0.04	0.04	0.04
37	Spathulenol	1578	1580	30.13	0.01	0.01	-	-
38	Caryophyllene oxide	1583	1586	30.34	0.14	0.10	0.13	0.13
39	ar-Turmerone	1665	1665	33.45	0.08	0.01	-	-
	Total				99.35%	99.4%	99.34%	99.45%

Grouped components (%).

Monoterpene hydrocarbons (Sr. No. 1–3, 5–7,9–12, 15, 17, 18).

Oxygen-containing monoterpenes (Sr. No,4,8,13–14, 16, 19–29).

Sesquiterpene hydrocarbons (Sr. No. 30–35).

Oxygen-containing sesquiterpenes (Sr. No. 36–39).

Data are mean ± standard error of three replications. ND, not detected. RIExp*, retention indices. RI values were obtained from NIST database and Adams (2001); RILit**, Retention indices taken from literature (12, 37); RT***, Retention time; D, distillation (%); a, Means followed by the same letter within a row are not significantly different according to Duncan’s multiple range test at *P* < 0.05.

**Fig. 12:**
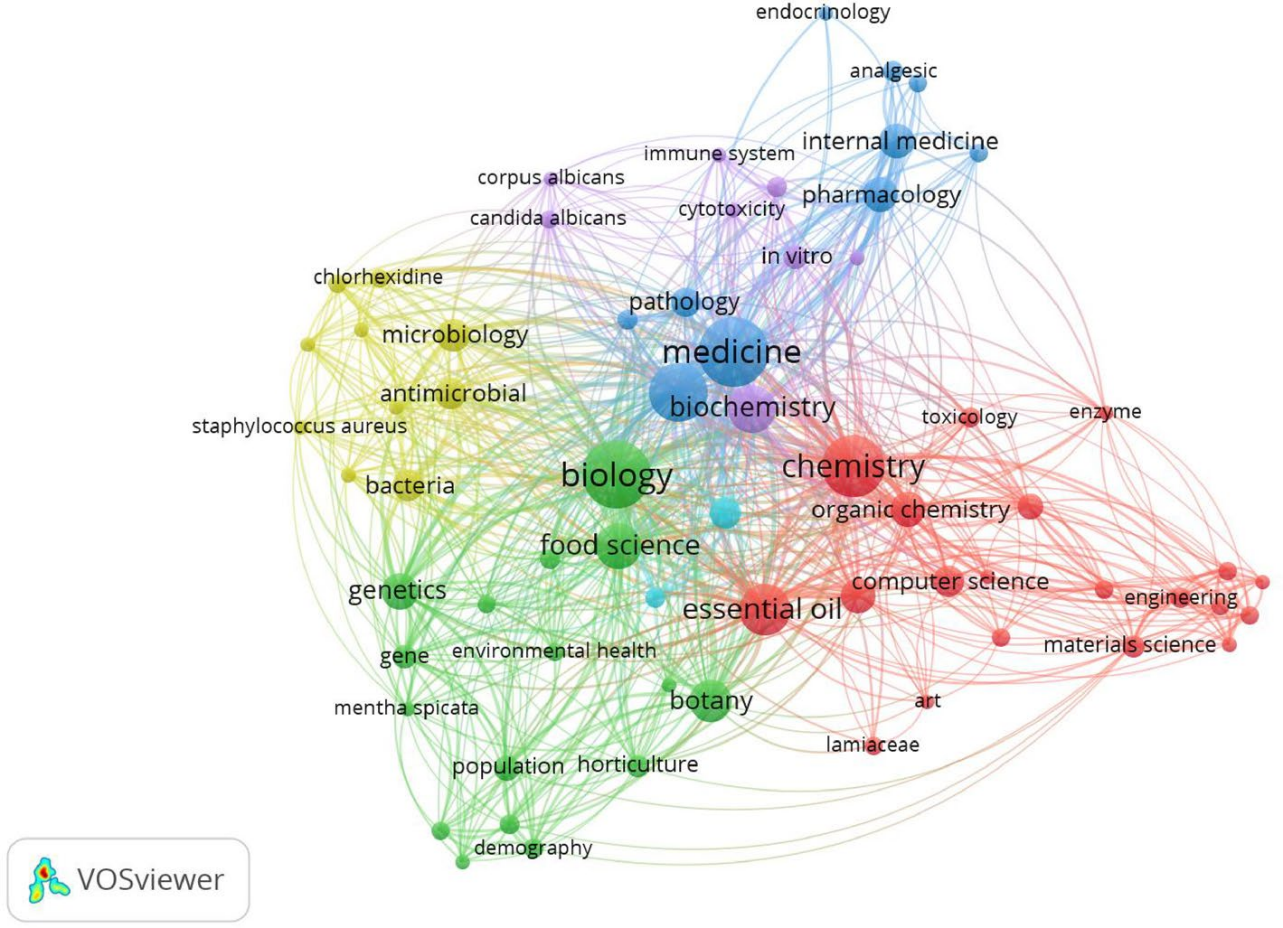
Co-occurrence analysis of the terms used more than 100 times as author and/or index keywords in essential oils research. The frequency of occurrence is represented by the size of the circle beneath each word. Different colors were employed to depict distinct clusters of highly related keywords, facilitating their categorization. The VOS viewer software was utilized to represent the term cloud, with data collected from the API database.

Essential oils are highly valued for their broad range of biological activities, including antimicrobial, antidiabetic, repellent, anticancer, antioxidant, and anti-inflammatory effects^25,26^. There is a growing trend toward using plant-based essential oils as natural alternatives to synthetic substances in promoting human health^27^ (Fig. 12). The storage of essential oils plays a crucial role in maintaining their quality^28^. The composition of essential oils may vary due to multiple influencing factors, including environmental conditions, harvest time, plant growth stages, and the methods used for extraction^29,30^. Additionally, environmental elements like light, temperature, and oxygen availability have been shown to alter the composition of essential oils during storage^31^. The degradation and instability of essential oils during storage are primarily due to these environmental influences^28^. Among these factors, temperature is considered one of the most significant determinants of essential oil quality^19,32,33^.

The essential oil profiles of *Melissa officinalis*,* Teucrium polium*,* Cuminum cyminum*,* Zhumeria majdae*,* and Thymus daenensis* were analyzed under varying storing conditions: refrigeration, freezing, and room temperature. Similar to previous research, our results indicated that compounds with lower boiling points were prone to a decrease in concentration at room temperature. However, after kept at lower temperatures, mainly at -20 °C, the essential oil compositions remained relatively stable, preserving their original integrity^33^. *Z. tenuior*, a medicinal plant from the mint family with diverse therapeutic applications, has an essential oil whose preservation under varying temperatures is not yet fully understood^34^. Studies have demonstrated its medicinal benefits, including treatment for hypertension, asthma, abscesses, respiratory and heart conditions, fever, digestive issues, and arrhythmias^35^. This plant exhibits a range of therapeutic properties, including relief for respiratory congestion, protection against colds, antibacterial and antioxidant effects, digestive cleansing, as well as anti-parasitic and immune-strengthening benefits^36^. Due to the presence of quercetin, combined with vitamin C, it is also suggested as a potential supplement for COVID-19 treatment^12^. The chief components of *Z. tenuior* essential oil include limonene, 1,8-cineole, and pulegone, which are valuable in the food industry^12^. Similarly, *Z. tenuior* essential oils are rich in pulegone, 1,8-cineole, limonene, α-pinene, and β-pinene. The research demonstrated that storing *Z. tenuior* essential oil at low temperatures, especially in refrigerators and freezers, led to minimal compositional changes and preserved its quality effectively. Earlier studies on the storage of essential oils from medicinal plants have similarly shown that refrigeration and freezing are the most effective methods for preserving oil integrity, aligning with the results of this research^33^. composition of *Z. tenuior*, a species with limited post-harvest data despite its ecological and pharmacological importance. The results not only clarify how storage affects chemical stability, but also contribute to evidence-based recommendations for preserving the value of endangered medicinal plants.

## Conclusion

Throughout the storage of essential oils, compounds with lower boiling points, especially monoterpene hydrocarbons, are particularly susceptible to loss. The results of the current study demonstrate that the highest retention of the main compound occurred when the essential oil of *Z. tenuior* was kept at 4 °C and − 20 °C. In contrast, storing the oil at room temperature over a three-month period resulted in a more significant decline in quality. These findings suggest that refrigeration and freezing better preserve the oil’s original quality compared to room temperature storage. In general, storing *Z. tenuior* volatile oils at lower temperatures stops the degradation of its components, ensuring minimal changes and better maintenance of its primary characteristics. These results may also be relevant for essential oils from other plants with similar chemical compositions. Additionally, this research provides valuable insights for producers and consumers in the pharmaceutical and cosmetic industries, offering practical guidance for optimal storage conditions. Overall, the study of storage methods for secondary herbal products, particularly critical oils, is an important area for more investigation, especially for oils composed of diverse chemical compounds.

## Data Availability

The data used in this study is openly available, and the data used is available upon request from the corresponding authors SH.N.
